# Multifunctional porous poly (L-lactic acid) nanofiber membranes with enhanced anti-inflammation, angiogenesis and antibacterial properties for diabetic wound healing

**DOI:** 10.1186/s12951-023-01847-w

**Published:** 2023-03-27

**Authors:** Hao Yu, Yijia Li, Yining Pan, Hongning Wang, Wei Wang, Xiaobin Ren, Hang Yuan, Ziru Lv, Yijia Zuo, Zhirong Liu, Wei Lin, Qingqing Yao

**Affiliations:** 1 National Engineering Research Center of Ophthalmology and Optometry, School of Ophthalmology & Optometry, Eye Hospital, Wenzhou Medical University, 270 Xueyuan Xi Road, Wenzhou, 325027 People’s Republic of China; 2 Institute of Stomatology, School and Hospital of Stomatology, Wenzhou Medical University, Wenzhou, 325027 China

**Keywords:** Porous PLA nanofiber membrane, Sulfated chitosan, Immunomodulation, Angiogenesis, Diabetic wound healing

## Abstract

With increased diabetes incidence, diabetic wound healing is one of the most common diabetes complications and is characterized by easy infection, chronic inflammation, and reduced vascularization. To address these issues, biomaterials with multifunctional antibacterial, immunomodulatory, and angiogenic properties must be developed to improve overall diabetic wound healing for patients. In our study, we prepared porous poly (L-lactic acid) (PLA) nanofiber membranes using electrospinning and solvent evaporation methods. Then, sulfated chitosan (SCS) combined with polydopamine-gentamicin (PDA-GS) was stepwise modified onto porous PLA nanofiber membrane surfaces. Controlled GS release was facilitated via dopamine self-polymerization to prevent early stage infection. PDA was also applied to PLA nanofiber membranes to suppress inflammation. In vitro cell tests results showed that PLA/SCS/PDA-GS nanofiber membranes immuomodulated macrophage toward the M2 phenotype and increased endogenous vascular endothelial growth factor secretion to induce vascularization. Moreover, SCS-contained PLA nanofiber membranes also showed good potential in enhancing macrophage trans-differentiation to fibroblasts, thereby improving wound healing processes. Furthermore, our in vitro antibacterial studies against *Staphylococcus aureus* indicated the effective antibacterial properties of the PLA/SCS/PDA-GS nanofiber membranes. In summary, our novel porous PLA/SCS/PDA-GS nanofiber membranes possessing enhanced antibacterial, anti-inflammatory, and angiogenic properties demonstrate promising potential in diabetic wound healing processes.

## Background

According to 2019 International Diabetes Federation data, an estimated 463 million adults had diabetes, and the numbers are expected to increase to over 700 million by 2045 [1]. With increased diabetes mellitus rates, the incidence of diabetic wounds, one of the most common diabetic complications, has been challenging in clinical settings owing to long treatment duration, high healthcare costs, high recurrence rates, and mortality [2, 3]. Generally, normal wound healing processes are characterized by typical overlap stages comprising hemostasis, inflammation, proliferation, and tissue remodeling [4, 5]. However, diabetic wound healing is hindered by chronic inflammation, ease of infection, and reduced neovascularization [6, 7].

Long-term inflammatory diabetic conditions generate reactive oxygen species (ROS) and inflammatory cytokine overexpression (such as interleukin-6 (IL-6), TNF-alpha (TNF-α) and IL-1beta (IL-1β)) [8]. Also, increased M1 macrophage polarization causes chronic inflammation and excessive inflammation hinders the transition from inflammation to proliferation stages. Hence, immunomodulating macrophage polarization toward M2 phenotypes (anti-inflammatory) to facilitate transition from inflammation to proliferation stages could effectively promote diabetic wound healing [9, 10]. In recent years, polydopamine (PDA) has attracted considerable attention in the wound healing field owing to its good anti-inflammatory activity, ROS scavenging ability, good tissue adhesion, and excellent cell affinity properties [8, 11, 12]. PDA contains biomaterials with rich reductive functional groups, such as catechol and amine groups, which help scavenge radical species [11, 13]. For example, Ma et al. showed that PDA-decorated microneedles inhibited ROS-induced inflammation, further promoting M2 macrophage polarization, suppressing wound inflammation, and facilitating wound healing [14]. Zhang et al. reported that PDA-treated titanium significantly reduced M1 macrophages by activating nuclear factor-κappaB signaling [15]. Additionally, due to plentiful phenol groups, PDA strongly adheres to tissues, including human skin [11]. Hence, PDA is an easy-to-modify substrate and provides anti-inflammatory microenvironments for wound healing.

Angiogenesis is a key process that accelerates diabetic wound healing by supplying oxygen and nutrients [16]. Typically, growth factors, such as vascular endothelial growth factor (VEGF) and basic fibroblast growth factor (bFGF), have been used to induce vascularization in diabetic wounds in clinical settings. However, growth factor-based therapy success is significantly hindered by high costs, high-dose requirements, relatively short half-life, and serious side effects [7]. Thus, new strategies need to be investigated. Recently, several studies suggested that M2 macrophages facilitated vascularization as they secreted angiogenic factors, such as platelet-derived growth factor (PDGF) and VEGF [17, 18]. Yu et al. reported that sulfated chitosan (SCS) had high affinity for VEGF, promoted VEGF binding to the VEGF receptor 2 (VEGFR2) and stimulated endothelial cell proliferation, migration, tubule networking, and promoted VEGF-mediated angiogenesis [19]. Studies also indicated that SCS induced macrophages toward the M2 phenotype via the interleukin-4 (IL-4) mediated Stat6 signaling pathway. Moreover, M2 macrophages enhanced macrophage trans-differentiation into fibroblasts [20, 21]. Therefore, SCS may suppress inflammation at early stages and promote neovascularization [20, 21].

Diabetic wounds are prone to bacterial infections because of constant exposure to external environments, which impedes wound treatment [22–24]. Therefore, strategies incorporating multifunctional scaffolds with antimicrobial, immunomodulatory, and angiogenic activities for diabetic wound healing must be promoted [25–27]. Although various wound dressing materials have been used for wound healing, nanofiber scaffolds that mimic both extracellular matrix (ECM) composition and structure have shown promising applications in skin regeneration [28, 29]. The electrospinning method is capable to produce nanofibers with diameters similar to those of natural ECM, and large specific electrospun nanofiber surface areas, together with high electrospun nanofibrous scaffold porosity, facilitate cell adhesion, proliferation, migration and differentiation [30–32]. Electrospun nanofibers with porous structures can also be used as drug carriers for drug release, thus inducing particular cell behaviors. Moreover, nanofiber porous structures increase the specific surface areas and also create opportunities for liquid and gas exchange to facilitate microenvironments for wound healing [1, 33]. Recently, polylactic acid (PLA), an FDA approved biopolymer, has attracted great attention in biomedical applications due to its good biocompatibility, biodegradability, relatively high mechanical properties and easy to process [34].

In this study, porous PLA nanofibers were prepared via electrospinning method by varying solvent ratios, PLA concentrations, and electric field intensity parameters. SCS was applied to these nanofibers to improve the hydrophilicity of PLA nanofiber membranes. SCS also drove macrophage polarization toward the M2 phenotype, thus suppressing inflammation and promoting neovascularization. PDA and the antibiotic gentamicin sulfate (GS) were decorated onto PLA/SCS nanofiber membranes via a PDA-assisted assembly strategy to obtain effective anti-inflammatory and antibacterial properties. PDA also facilitated strong fibrous membrane adherence to wounded tissues via electrostatic interactions and covalent bonds. In summary, porous PLA nanofiber membranes with angiogenic, anti-inflammatory, and antibacterial properties with possible applications in diabetic wound healing were developed.

## Methods

### Materials

PLA and GS were purchased from Sigma-Aldrich. Chitosan (CS, 95% deacetylated powder) and dopamine hydrochloride were provided by Macklin Biochemical Co., Ltd. Chlorosulfonic acid, dichloromethane (DCM) and N, N-dimethylformamide (DMF) were purchased from Aladdin Reagent Co. (Shanghai, China).

### SCS fabrication and characterization

CS was dissolved in formamide at 50 ℃ with stirring to generate a 2wt% homogeneous CS solution. Then, a chlorosulfonic acid/DMF (volume ratio = 4:1) mixed solution was dropped into the CS solution and reacted at 50 ℃ for 2 h. This solution against deionized water (DI) for 96 h and replaced the DI water twice a day. Finally, the resultant solution was stored at − 80 ℃ overnight, and then freeze dried for 72 h.

To determine CS and SCS chemical composition, Fourier-transform infrared spectroscopy (FTIR, Nicolet, USA) was performed. Samples were prepared by mixing 1 mg of powder with 200 mg of KBr powder and by pressing into pellets. The FTIR spectra were collected in the 4000–400 cm^−1^ range in the transmission mode with a resolution of 4 and scans of 32.

To confirm the CS sulfonation, proton nuclear magnetic resonance (^1^H NMR) measurements were performed. CS and SCS powders were dried in the oven overnight at 80 ℃, then dissolved in deuterium oxide (D_2_O) at the concentration of 1 mg/mL, and ^1^H NMR data were collected at 37 °C and analyzed using a MestReNova software.

### In vitro angiogenesis assays and immune response of SCS

The influence of SCS on the angiogenic and immune responses of macropahges, RT-PCR was performed as previously reported [34]. For the SCS, Raw 264.7 cells (5 × 10^4^) were seeded on the 24-well plate and cultured overnight, after that SCS/culture medium was added. Total RNA was extracted from macrophages using the GeneJET™ RNA Purification Kit and reverse transcribed into cDNA using the High Capacity cDNA Reverse Transcript kit according to the manufacturer’s instructions. Angiogenic marker (hypoxia-inducible factor 1-alpha (HIF-1α) and VEGF and inflammatory gene marker expression (tumor necrosis factor-α (TNF-α), IL-1β, IL-6, IL-4, IL-10, and Arg-1) were quantified using RT-PCR. β-actin was the housekeeping gene. The primer sequences are listed in Table 1.

**Table 1:** RT-PCR primer sequences

Gene name	Forward primer	Reverse primer
HIF-1α VEGF	ACCTTCATCGGAAACTCCAAAG GTCCTCTCCTTACCCCACCTCCT	CTGTTAGGCTGGGAAAAGTTAGG CTCACACACACAGCCAAGTCTCCT
IL-6	TGTGTTTTCCTCCTTGCCTCTGAT	TGCTGCCTAATGTCCCCTTGAAT
IL-1β	TGTGTTTTCCTCCTTGCCTCTGAT	TGCTGCCTAATGTCCCCTTGAAT
TNF-α	CTTGTTGCCTCCTCTTTTGCTTA	CTTTATTTCTCTCAATGACCCGTAG
IL-4	GCGTGCTTGCTGGTTCT	GTCCTGGGCTCCCTCTC
Arg-1	GGCAACCTGTGTCCTTTCTCCT	CCCAGCTTGTCTACTTCAGTCATG
IL-10	GGAAGACAATAACTGCACCCACT	CAACCCAAGTAACCCTTAAAGTCC

### Preparation and characterization of PLA nanofiber membranes

PLA was dissolved in DCM/DMF mixtures (volume ratios of 9/1, 8/2, and 7/3) at 10 wt% and the solution transferred to a 5 mL syringe for electrospinning. The parameters were as follows: flow rate = 1 mL/h, collecting distance = 20 cm, and voltage = 20 kV.

Porous PLA fiber membrane morphologies were observed using scanning electron microscopy (SEM, Prox, Phenom) at 10 kV accelerating voltage. Samples were sputter-coated with gold for 40 s. Fiber diameters and distributions were measured using SEM images by ImageJ (win64, National Institutes of Health, USA), and more than 100 fibers were randomly were analyzed per sample.

### Preparation and characterization of PLA-based nanofiber membranes

To prepare PLA/SCS nanofiber membranes, PLA nanofiber membranes were immersed in SCS solution (10 mg/mL) for 1 h and dried overnight in a fume hood.

To prepare PLA/SCS/PDA nanofiber membranes, PLA/SCS nanofiber membranes were immersed in Tris-buffer (pH 8), dopamine hydrochloride (10 mg/mL) was then added into the solution. After that, the mixed solutions were reacted overnight with stirring.

To prepare PLA/SCS/PDA-GS nanofiber membranes, the prepared PLA/SCS nanofiber membranes were mixed with the Tris-buffer, dopamine hydrochloride and GS were added into the solution and reacted overnight with stirring.

### Wettability tests

Porous PLA, PLA/SCS, PLA/SCS/PDA, and PLA/SCS/PDA-GS nanofiber membranes with a dimension of 2 × 2 mm were subjected to the contact angle test. The wettability of the prepared samples was determined using a water contact angle instrument (OCA25, Dataphysics, Germany). Three parallel tests were measured on each nanofiber membrane groups.

### Swelling tests

Equilibrated swelling ratios (ESRs) of porous PLA, PLA/SCS, PLA/SCS/PDA, and PLA/SCS/PDA-GS nanofiber membranes were measured in phosphate buffered saline (PBS) solution. The nanofiber membranes were first weighed (dry weight (M_0_)) and then transferred into PBS at 37 ℃. The nanofiber membranes were removed carefully at different time points and weighed after removing the excess solution (M_W_) until the swelling equilibrium was reached.

ESRs were calculated as follows:

$${\text{ESRs }} = \, \left( {{\text{M}}_{{\text{W}}} - {\text{M}}_{0} } \right)/{\text{M}}_{0} \times { 1}00\%$$

where M_0_ and M_w_ = membrane weights under dry and swollen conditions, respectively. Three repeated measurements were performed.

### In vitro GS release

PLA/SCS/PDA-GS nanofiber membranes were used in the in vitro GS release study. The prepared nanofiber membranes were immersed in Dulbecco's phosphate-buffered saline (DPBS) medium at 37 ℃ and agitated at 90 rpm. At different time points, half the supernatant was collected and the same volume of fresh DPBS was added. After 7 days of release, the released GS amounts were determined using UV-Vis spectrophotometry (ZF-20D, Shanghai, China), as described elsewhere [35].

### In vitro cell culture

Mouse macrophages cell line (Raw 264.7) and human umbilical vein endothelial cells (HUVECs) were purchased from the Procell Life Science&Technology Company (Wuhan, China). Raw 246.7 cells and HUVECs were cultured with DMEM high-glucose. All medium supplemented with 10% FBS and 1% penicillin/streptomycin (P/S, Beyotime). Moreover, all cells were cultured in a humid incubator at 37 °C and a CO_2_ level of 5%.

#### In vitro cytotoxicity assays

The morphologies of Raw264.7 cells on porous PLA, PLA/SCS, PLA/SCS/PDA, and PLA/SCS/PDA-GS nanofiber membranes were determined using the Calcein-AM and propidium iodide (PI) stains, which labeled live and dead cells, respectively. At each incubation time point, the culture medium was removed and the cells were washed with three times in DPBS. Calcein-AM and PI in DPBS were then added to the 24-well plates and incubated for 15 min at room temperature. Fluorescence images were recorded using a laser scanning microscope (DFC7000 GT DMi8, Leica, Germany).

Nanofiber membrane cytotoxicity was quantitatively analyzed using CCK8 assays (Dojindo Molecular Technologies, Inc.) according to the manufacturer’s instruction. Briefly, porous PLA, PLA/SCS, PLA/SCS/PDA, and PLA/SCS/PDA-GS nanofiber membranes were placed in 24-well plates, 5 × 10^4^ Raw264.7 cells were then seeded onto the prepared nanofiber membranes. After 1 and 3 days of culture, the culture medium was removed, 10% CCK8/culture medium was added, the cells were incubated for 2 h, and the absorbance was measured at 450 nm on a microplate reader (Infinite M200, Tecan, USA). PLA nanofiber membranes acted as controls, and the cell viability was expressed as percentages relative to the control group.

#### Real-time polymerase chain reaction (RT-PCR)

To determine the influence of the PLA-based nanofiber membrane on macrophage angiogenic and immune responses, RT-PCR was performed. For PLA-based samples, Raw 264.7 cells (5 × 10^4^) were seeded on PLA, PLA/SCS, PLA/SCS/PDA, and PLA/SCS/PDA-GS nanofiber membranes and cultured for 1 and 3 days. And gene expression data were acquired.

#### In vitro antibacterial culture

The antibacterial activities of porous PLA, PLA/SCS, PLA/SCS/PDA, and PLA/SCS/PDA-GS nanofiber membranes were studied using the Gram-positive bacteria *Staphylococcus aureus* (*S. aureus*) (ATCC 6538). Same volume of *Staphylococcus aureus*-containing suspensions were spread on the agar surface for inoculation. Zones of inhibition (ZOI), shake-flask culture, and bacterial live/dead staining tests were carried out to determine the anti-adhesive and bactericidal functions of nanofiber membranes toward *S. aureus*.

#### ZOI assays

*S. aureus* suspension (1.0 × 10^6^ CFU/mL) were uniformly spread onto nutrient agar, afterward, the prepared PLA, PLA/SCS, PLA/SCS/PDA, and PLA/SCS/PDA-GS nanofiber membranes were placed on the Petri dishes and incubated at 37 ºC for 24 h. ZOIs were measured using a perpendicular caliper. Three parallel samples were measured to assess the antimicrobial activity of the nanofiber membrane.

#### Bacteria killing tests

Bacteria killing properties were studied by immersing the nanofiber memberanes into bacterial solutions in shake-flask tests. After immersing for 15 min, bacterial solutions had homogeneous coated the membranes, which were placed onto solid agar and incubated, after which bacterial numbers were counted. The survival rate was defined as the percentage of bacteria relative to the initial total number in the suspension.

#### Live/dead bacterial staining

*S. aureus* suspensions (1.0 × 10^5^ CFU/mL) were seeded onto the PLA, PLA/SCS, PLA/SCS/PDA, and PLA/SCS/PDA-GS nanofiber membranes and co-cultured for 24 h. Samples were then stained using a live/dead BacLight bacterial viability kit (L-7012, Invitrogen) according to the manufacturer’s instructions. Bacterial morphology was observed by a fluorescence microscopy (Zeiss, Germany).

#### Statistical analysis

The One-way ANOVA with Tukey’s correction (compare among groups) and Student’s t-tests (between two groups) were used to calculate statistical significance. A value of p < 0.05 was considered to be statistically significant. All the data are presented as the mean ± standard deviation (SD).

## Results and discussion

### SCS synthesis and characterization

**Fig. 1:**
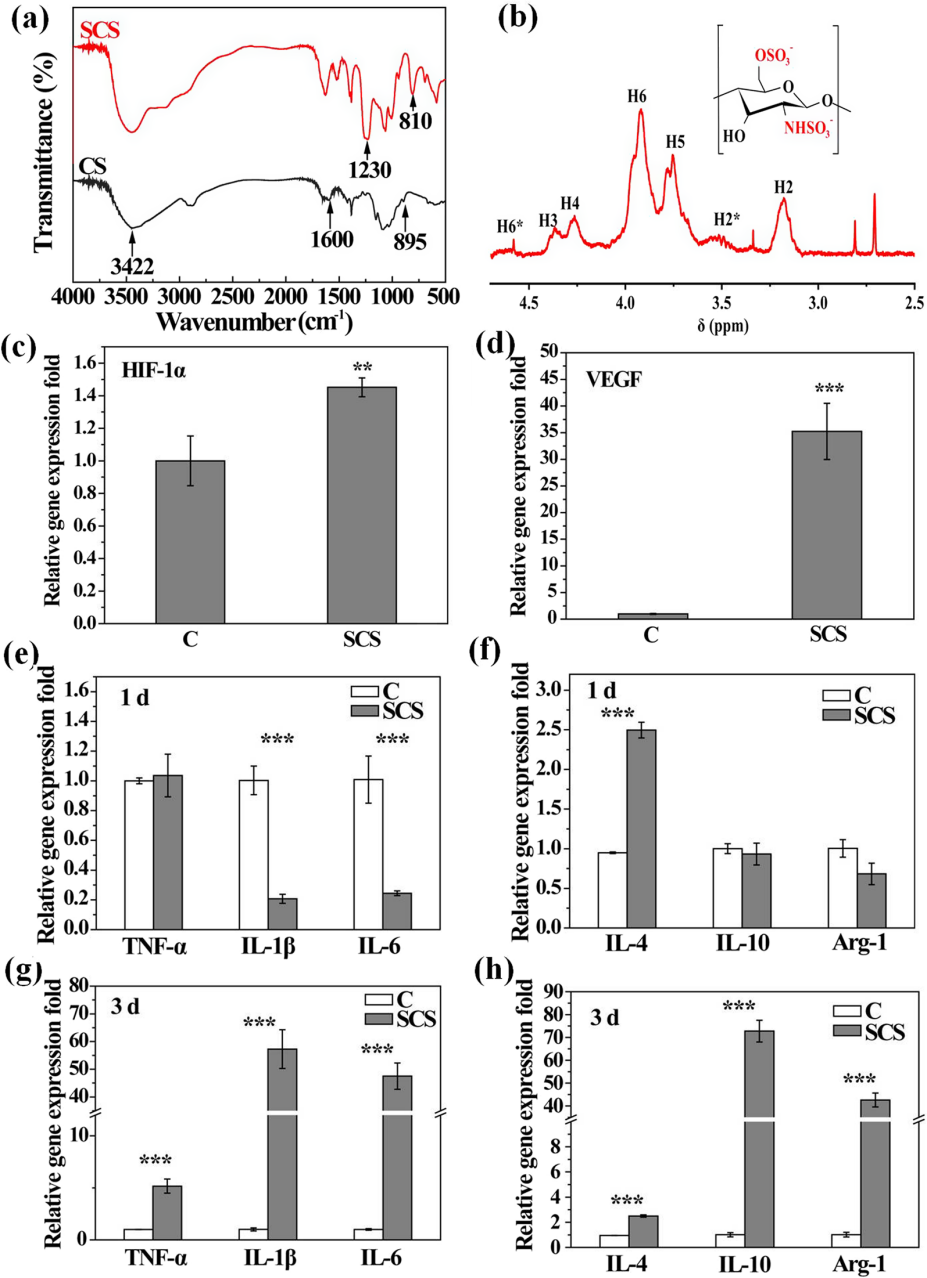
**a** CS and SCS FTIR spectra; **b** SCS ^1^H NMR spectrum; **c** HIF-1α and **d** VEGF gene expression in Raw.264.7 cells in SCS/culture medium for 24 h; relative gene expression of **e**, **g** TNF-α, IL-1β, IL-6 and **f**, **h** IL-4, IL-10, Arg-1 of Raw 264.7 cells in SCS/culture medium for 24 h and 48 h of culture, respectively

To analyze CS sulfation, FTIR and ^1^H NMR were measured (Fig. 1a, b). The peak around 3422 cm^−1^ was belonged to the overlapping of the stretching vibration of O–H and the stretching vibration of N–H groups of chitosan. The peak at 895 cm^−1^ was the stretching vibration peak of C-O. And the peak around 1600 cm^−1^ in chitosan was attributed to the bending vibration peak of protonated amino group. When compared with CS, the FTIR spectra of SCS showed the new bands at approximately 810 cm^−1^ and 1230 cm^−1^, which were attributed to C-O-S and O = S = O group stretching vibrations, respectively. Moreover, peaks at 3.3 ppm and 4.6 ppm in SCS ^1^H NMR spectra of SCS confirmed that the sulfonation had occurred positions in C2 and C6 of CS. These data suggest successful CS sulfation.

**Fig. 2:**
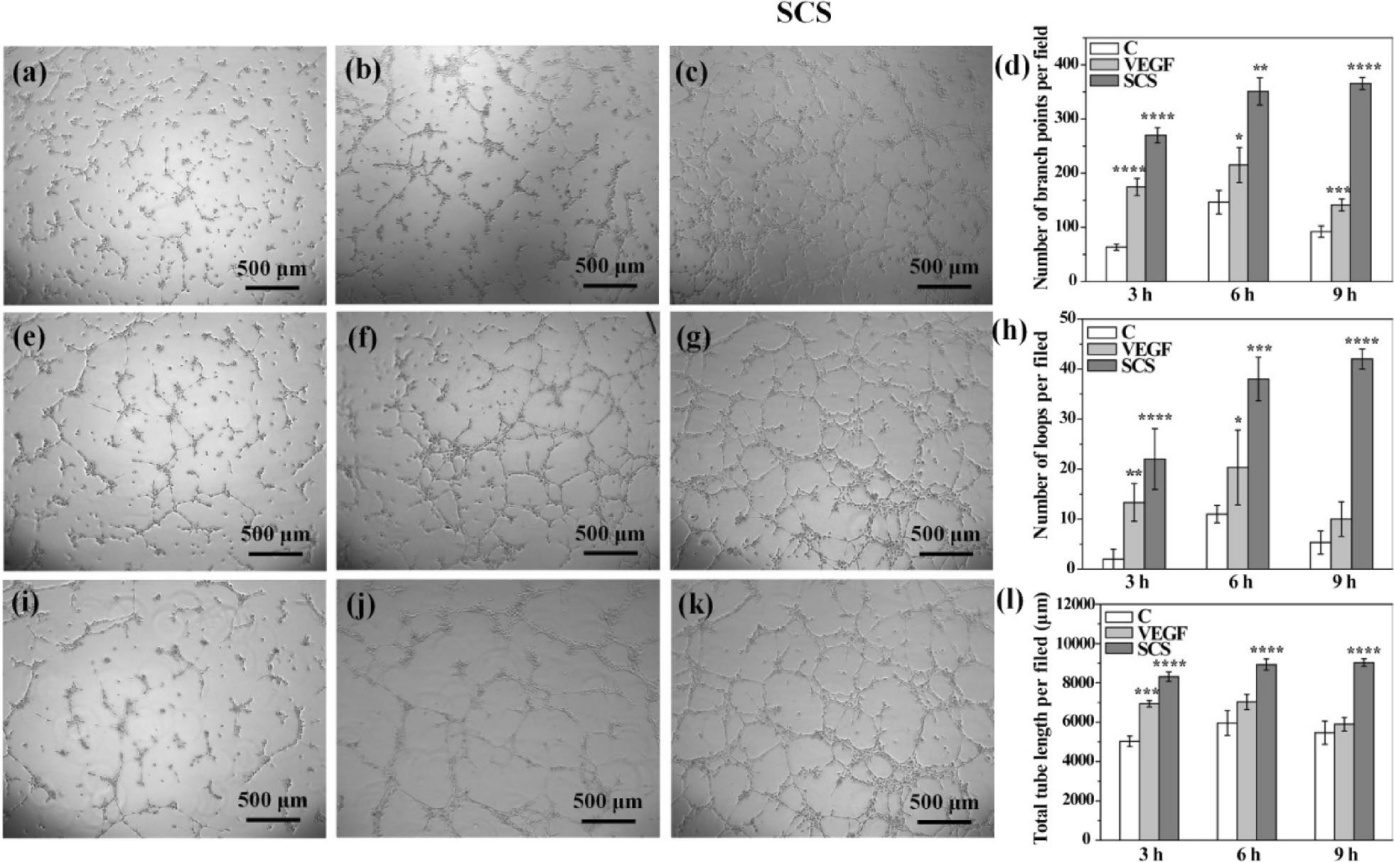
Representative bright light images showing the capillary networks after 3 h, 6 h, 9 h of (**a**, **e**, **i**) Control, (**b**, **f**, **j**) VEGF and (**c**, **g**, **k**) SCS treatment, respectively; quantitative analysis of sprouted HUVECs on (**d**) number of branch points per field, (**h**) the number of loops per field and (**l**) total capillary tube length per field

Angiogenesis is a complex and coordinated process involving multiple-factors [36, 37]. It remains a challenge to induce vascularization in engineered tissues by delivering just one growth factor (e.g., VEGF or PDGF-BB). As a key upstream transcription factor, HIF-1α plays important roles in bone tissue engineering via VEGF [38, 39] and stromal cell-derived factor 1 (SDF-1) generation to upregulate the VEGF-induced vascularization and SDF-1 induced progenitor cells recruitment [40, 41]. HIF-1α and VEGF gene expression levels in Raw 264.7 cells were significantly increased after 24 h of SCS treatment (Fig. 1c, d). The pro-angiogenic potential of SCS in human umbilical vein endothelial cells (HUVECs) was also evaluated using capillary tube formation assays (Fig. 2). The exogenous VEGF- (6 ng/mL) treated group was used as a positive control. Capillary-like networks were formed on VEGF- and SCS-treated HUVECs after 3 h of treatment, whereas few capillary tubes were observed in the control group (***p < 0.001). All branch points, loops per field, and total capillary tube length per field were increased in VEGF and SCS-treated groups in the first 6 h. However, at 9 h, capillary-like networks no longer increased in the VEGF treated group, but more capillary-like networks were formed in the SCS treated group. Thus, SCS alone promoted capillary tube formation without exogenous VEGF addition, with higher enhanced capabilities when compared with the VEGF-treated group. Moreover, SCS effects were was longer than VEGF effects, which could be attributed to the short half-life of VEGF. Therefore, SCS alone promoted angiogenesis in HUVECs and induced pro-angiogenic factor expression in macrophages without exogenous VEGF addition. When compared with VEGF, SCS was easily available, cheap, had low toxicity, and was stable. In the future, SCS may be used as a VEGF substitute in clinical settings.

To study the influence of SCS on the macrophage polarization, pro-inflammatory and anti-inflammatory gene expression was investigated using RT-PCR. After 1 day of SCS treatment, M1 phenotype-related genes (IL-1β and IL-6) were decreased, whereas the M2 phenotype-related gene IL-4 was increased. Interestingly, after SCS incubation for 3 days, both M1 (TNF-α, IL-1β, and IL-6) and M2 phenotype-related genes (IL-4, IL-10, and Arg-1) were dramatically increased. As Shen et al. reported, some macrophages participate in proliferation processes during diabetic wound healing, and M1/M2 double-positive macrophages can benefit macrophage trans-differentiation to fibroblasts [21]. Therefore, SCS appears to regulate macrophages toward an anti-inflammatory phenotype and potentially promote macrophage trans-differentiation into fibroblasts.

### Synthesis and characterization of PLA-based nanofiber membranes

**Fig. 3:**
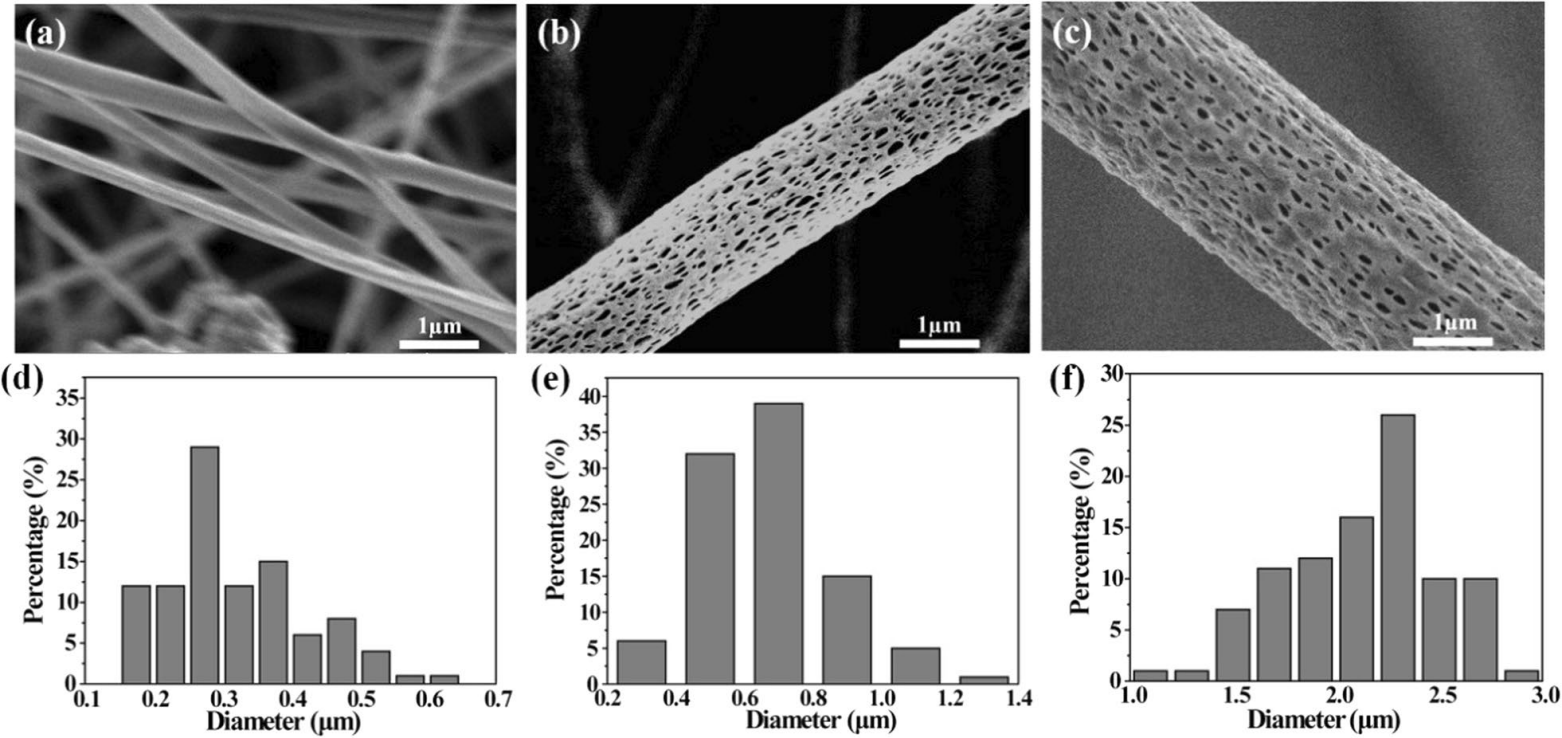
SEM images showing porous PLA nanofibers prepared with different mixed solvent ratios **a** DCM/DMF = 7/3, **b** DCM/DMF = 8/2 and **c** DCM/DMF = 9/1 (Scale bar = 1 μm); Porous PLA nanofiber diameters prepared using different solvent ratios **d** DCM/DMF = 7/3, **e** DCM/DMF = 8/2 and (f) DCM/DMF = 9/1

Our preliminary data showed that the mixture solvent ratios play the leading role in PLA nanofiber porous structures of PLA nanofibers when compared with the PLA concentrations, electric field intensity, and liquid flow rates (data not shown). Hence, the effects of solvent ratios on porous PLA nanofiber morphologies and corresponding diameter distribution were investigated. In the present work, a mixture of solvents with ratios of 7:3, 8:2 and 9:1 was performed. The SEM images showed that when DCM: DMF ratio varied from 7:3 to 9:1, PLA nanofiber porous structures were formed and nanofiber diameters increased stepwise (Fig. 3). Moreover, PLA nanofiber diameters were more uniform in the DCM: DMF 8:2 ratio when compared with 9:1 ratio. Upon organic solvent evaporation, pores were formed on PLA nanofiber surfaces, and owing to evaporation rate differences between DCM and DMF, increased DCM ratios enhanced the pore forming rates. Thus, the DCM: DMF ratios of 8:2 was selected as the optimal parameter to prepare electrospun porous PLA nanofiber membranes and used for subsequent studies.

**Fig. 4:**
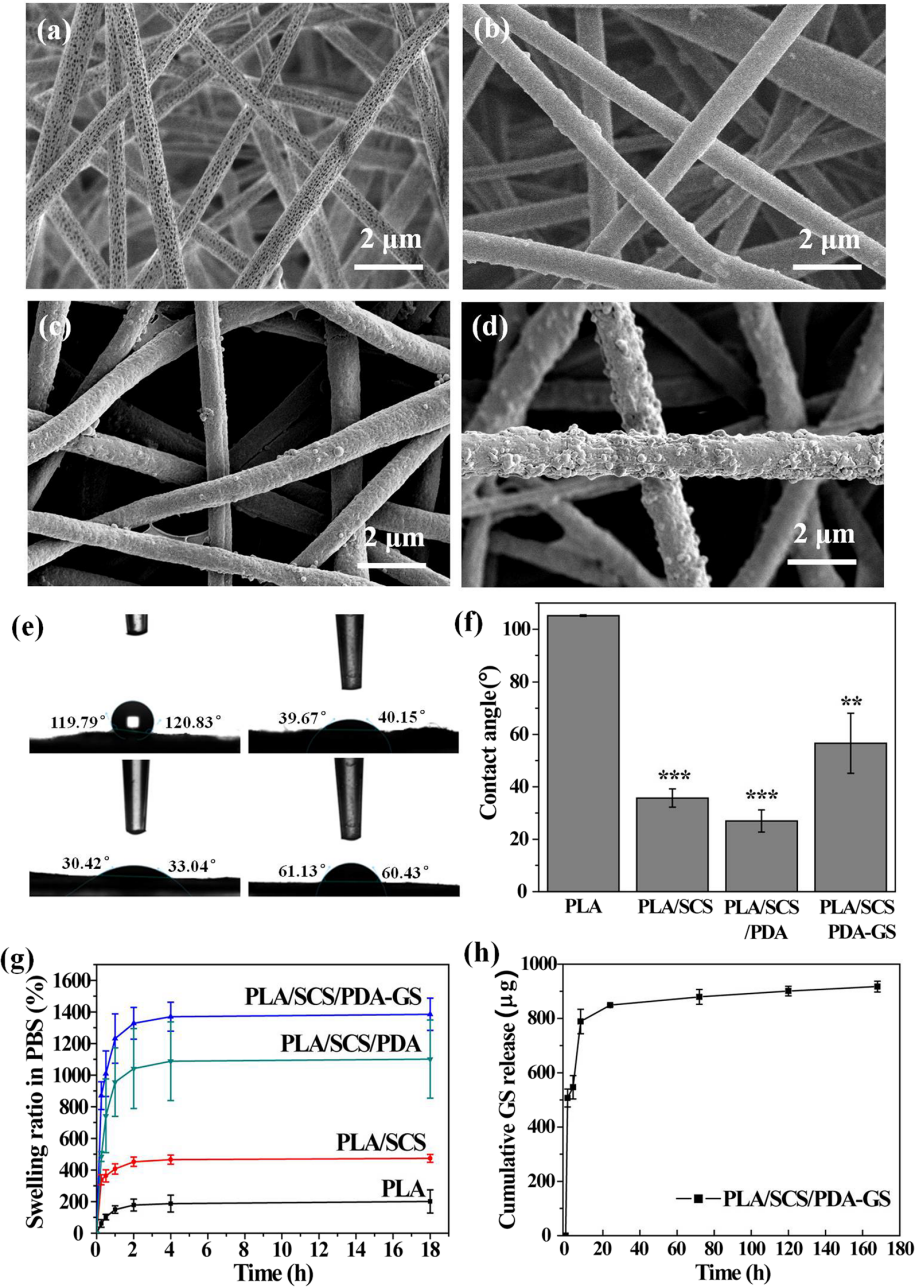
SEM images showing **a** porous PLA, **b** PLA/SCS, **c** PLA/SCS/PDA, and **d** PLA/SCS/PDA-GS nanofiber membranes, **e** Contact angle images of PLA, PLA/SCS, PLA/SCS/PDA and PLA/SCS/PDA-GS membranes; **f** Quantitative contact angle data of PLA, PLA/SCS, PLA/SCS/PDA and PLA/SCS/PDA-GS membranes; **g** swelling behaviors of PLA, PLA/SCS, PLA/SCS/PDA and PLA/SCS/PDA-GS membranes immersed in PBS solution for 18 h; **h** GS release profiles from PLA/SCS/PDA-GS membranes in PBS solution. Data are expressed as mean ± SD (n = 3)

Morphological changes in PLA nanofiber membranes after SCS, PDA, and GS modification were measured using SEM. After SCS modification, relatively smooth surfaces and increased PLA nanofiber diameters were observed, and the porous PLA nanofiber structures had disappeared (Fig. 4a, b). The diameter of PLA nanofiber was increased from 590 ± 120 nm to PLA/SCS (800 ± 300 nm), PLA/SCS/PDA (830 ± 170 nm) and PLA/SCS/PDA-GS (970 ± 230 nm). With self-aggregated PDA on the PLA/SCS nanofiber membrane surface, granular-like morphology was formed (Fig. 4c). Also, PLA/SCS nanofiber roughness and diameters were increased with the addition of GS into dopamine before the self-aggregation of PDA (Fig. 4(d)). PLA hydrophilicity was improved after SCS coating, and the PLA nanofiber membrane contact angle was decreased from 110° to 39° (Fig. 4e). No significant differences were observed between PLA/SCS and PLA/SCS/PDA nanofiber membranes, which indicates that PDA modifications had not altered the hydrophobicity of PLA/SCS nanofiber membranes. However, the contact angle of PLA/SCS/PDA-GS nanofiber membranes was increased due to GS hydrophobicity properties (Fig. 4f). The swell behaviors of PLA, PLA/SCS, PLA/SCS/PDA, and PLA/SCS/PDA-GS nanofiber membranes were examined in PBS (Fig. 4g). All the nanofibers membranes reached equilibrium after 4 h, and the swelling ratios were increased from PLA (ca. 200%) to PLA/SCS (ca. 440%), PLA/SCS/PDA (ca. 1180%), and PLA/SCS/PDA-GS (ca. 1380%). GS release profiles from PLA/SCS/PDA-GS nanofiber membranes are shown in Fig. 4h. An initial burst release was observed in the first 12 h, which was followed by a sustained release over 7 days. Thus, the efficient initial GS release from PLA/SCS/PDA-GS nanofiber membranes was beneficial to prevent early-stage wound healing infection.

### In vitro cell viability and pro-angiogenic marker gene expression

**Fig. 5:**
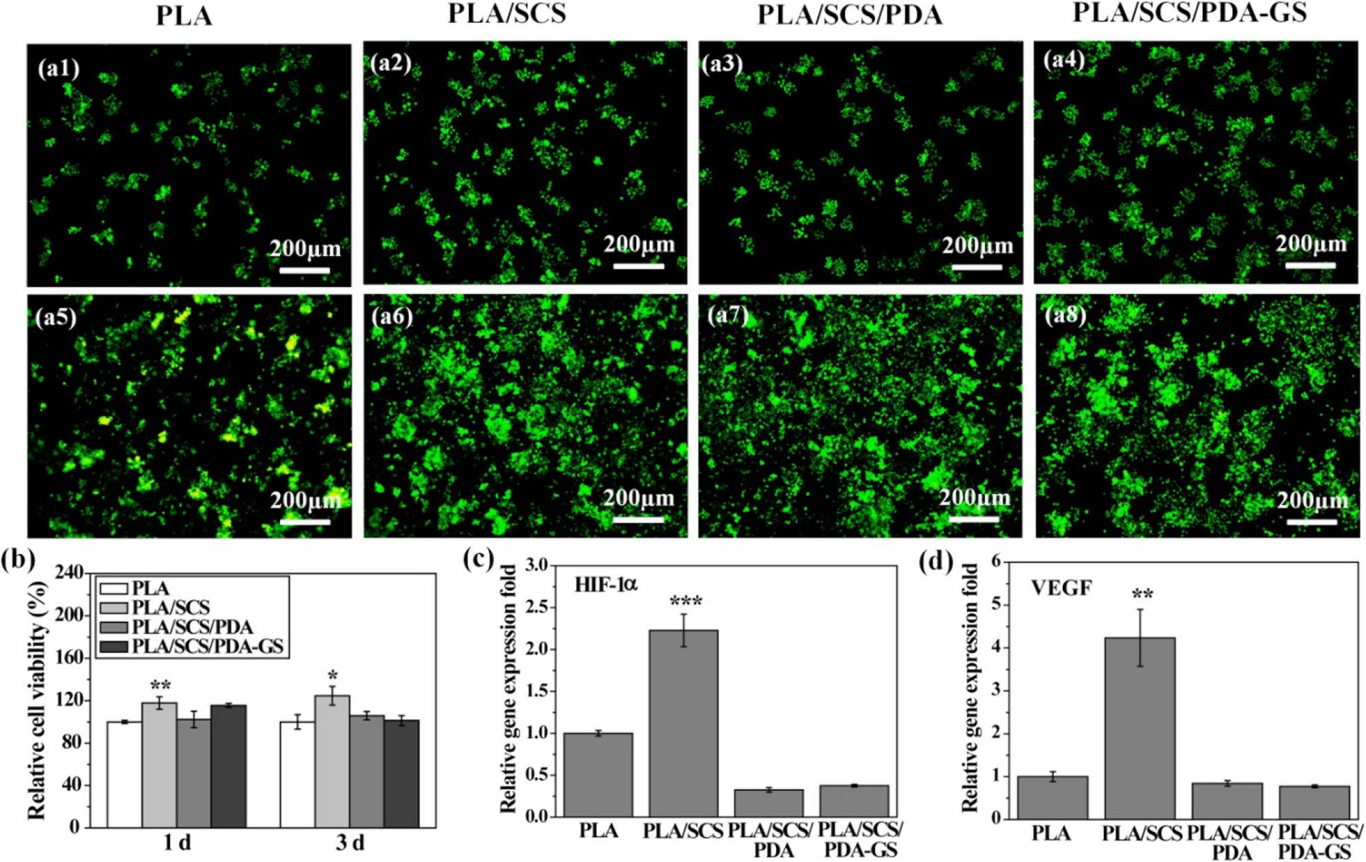
Fluorescence images of Raw 264.7 cells cultured on (**a1**, **a5**) PLA, (**a2**, **a6**) PLA/SCS, (**a3**, **a7**) PLA/SCS/PDA and (**a4**, **a8**) PLA/SCS/PDA-GS nanofiber membranes after 1 and 3 days of culture, respectively; **b** cell viabilities on PLA, PLA/SCS, PLA/SCS/PDA and PLA/SCS/PDA-GS nanofiber membranes after 1 and 3 days of culture; **c** HIF-1α and **d** VEGF gene expressions in Raw.264.7 cells after cultured in PLA, PLA/SCS, PLA/SCS/PDA and PLA/SCS/PDA-GS nanofiber memebranes for 24 h

The Live/dead Raw 264.7 cell staining of all nanofiber membranes is shown in Fiugre 5a1-a8. Raw 264.7 cells attached to and spread well on the porous nanofiber membranes, with cells proliferating well on day 3 when compared with day 1. Nanofiber membrane cytotoxicity levels are shown in Fig. 5b. Consistent with live/dead staining data, no obvious cell toxicities were detected on all four nanofiber membranes after 1 and 3 days of culture. Interestingly, after both 1 and 3 days of culture, the PLA/SCS group exhibited higher cell viability when compared with the PLA group, whereas no significant differences were obseverd among PLA, PLA/SCS/PDA and PLA/SCS/PDA-GS groups. Thus, all porous PLA-based nanofibers were suitable for cell growth, and SCS may be beneficial for cell proliferation.

We previously data showed that SCS alone induced the pro-angiogenic factor (HIF-1α and VEGF) expression in Raw 264.7 cells (Fig. 1c, d). Our in vitro gene expression data demonstrated that the PLA/SCS group expressed higher HIF-1α and VEGF levels when compared with the PLA group after 1 day of culture. Thus, after decorating the porous nanofiber membranes with SCS, the pro-angiogenic function of SCS was maintained. However, both PLA/SCS/PDA and PLA/SCS/PDA-GS groups did not increase HIF-1α and VEGF expression. These might have be due to a PDA layer forming on the PLA/SCS nanofiber membrane surface, and also more time might have been required for SCS to release and induce pro-angiogenic gene expression. Hyperglycemia in diabetes mellitus will cause endothelia dysfunction and damage the activity of VEGF, ultimately lead to impairment of neovascularization [42, 43]. In this work, SCS-containing PLA nanofiber membranes showed ability to increase endogenous VEGF expression and promote angiogenesis in HUVECs, and these will promote diabetic wound healing.

### Macrophage immune responses to porous PLA-based nanofiber membranes

**Fig. 6:**
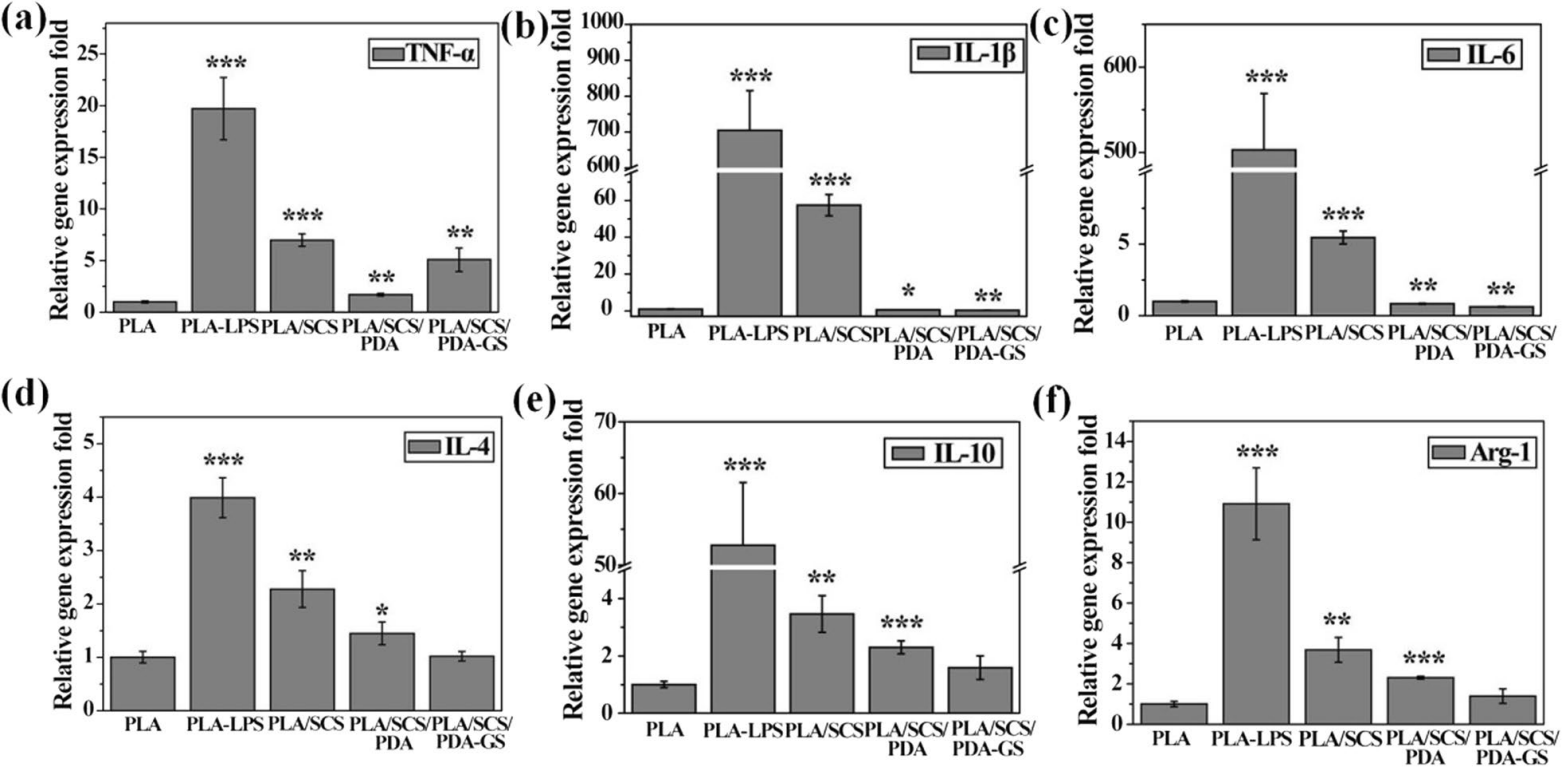
The relative gene expression levels of TNF-*α*, IL-1β, IL-6, IL-4, IL-10, and Arg-1 of Raw 264.7 cells in PLA, PLA/SCS, PLA/SCS/PDA and PLA/SCS/PDA-GS membranes after 24 h of culture

**Fig. 7:**
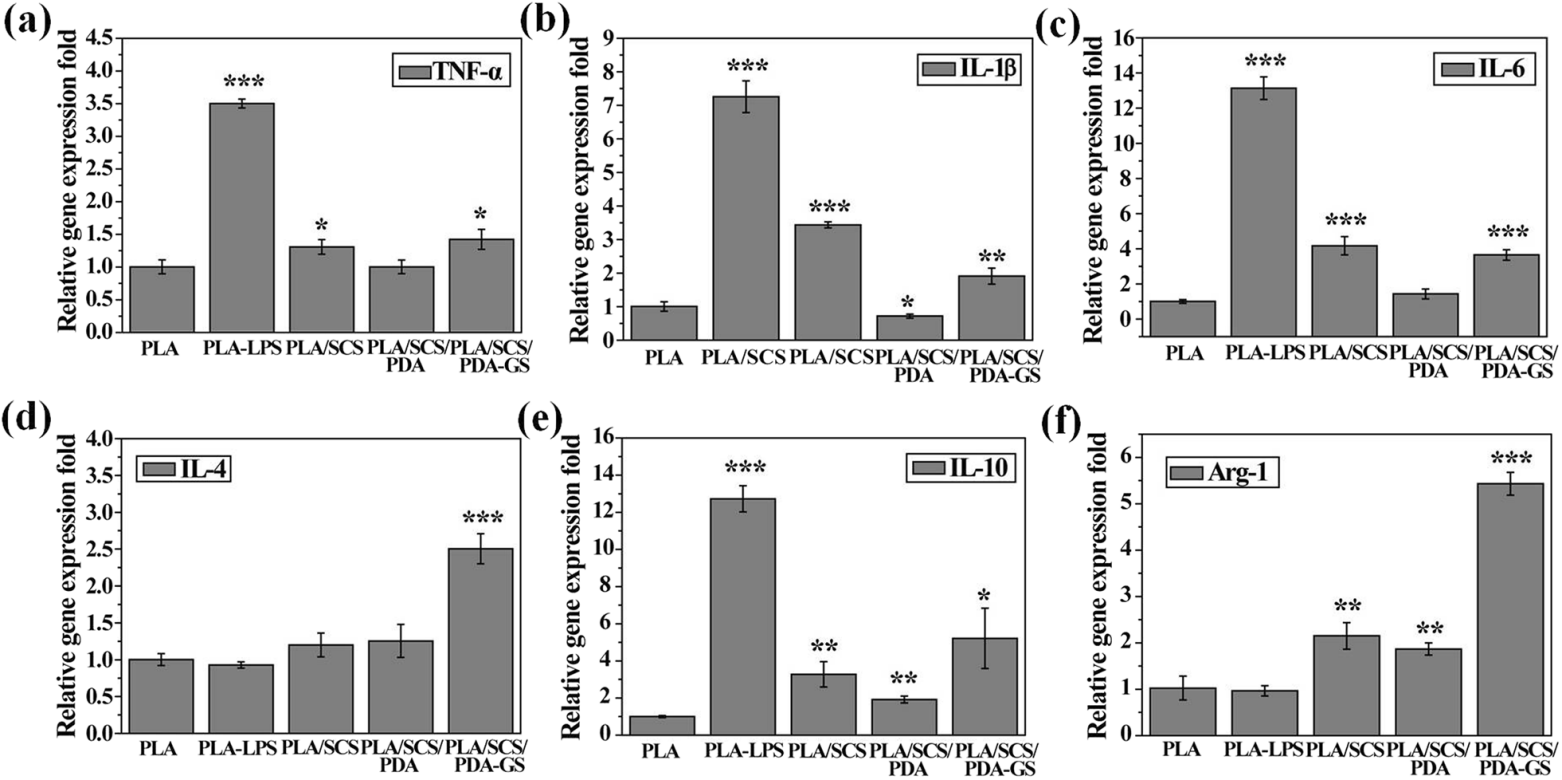
The relative gene expression levels of pro-inflammatory genes (TNF-*α*, IL-1β and IL-6) and anti-inflammatory genes (IL-4, IL-10 and Arg-1) of Raw 264.7 cells in PLA, PLA/SCS, PLA/SCS/PDA and PLA/SCS/PDA-GS membranes after 48 h of culture

To investigate the influence of porous PLA-based nanofiber membranes on the polarization of Raw 264.7 cells, RT-PCR was carried out. Lipopolysaccharide (LPS, 500 ng/mL), a potent activator of the inflammatory response, was used to stimulate macrophage polarization toward the proinflammatory M1 phenotype. After 24 h coculture (Fig. 6), LPS markedly increased pro-inflammatory gene expression (TNF-α, IL-1β and IL-6) (***p < 0.001), with PLA/SCS, PLA/SCS/PDA, and PLA/SCS/PDA-GS groups showing significantly reduced the TNF-α, IL-1β and IL-6 expression, especially PDA-containing groups. Notably, PLA/SCS and PLA/SCS/PDA groups showed elevated anti-inflammatory gene expression (IL-4, IL-10 and Arg-1). Forty-eight hours after the LPS stimulation (Fig. 7), similar results were observed for pro-inflammatory gene expression (TNF-α, IL-1β and IL-6) in all nanofiber groups. PLA/SCS and PLA/SCS/PDA groups showed increased anti-inflammatory gene expression, whereas GS-containing groups dramatically promoted anti-inflammatory gene expression.

Long-term inflammatory environment in diabetic wounds impaired macrophage phenotype transition from inflammatory (M1) to anti-inflammatory (M2) status, which will also impede the trans-differentiation of macrophages into fibroblasts. Moreover, M2 macrophages also play a pivotal role in revascularization owing to their ability to release angiogenic factors, such as VEGF and PDGF. Our cell tests data demonstrated that PLA/SCS/PDA-GS nanofiber membranes were able to regulate the macrophage inflammatory responses, forming new vessels, thus promoting diabetic wound healing.

### Antibacterial activity of porous PLA-based nanofiber membranes

**Fig. 8:**
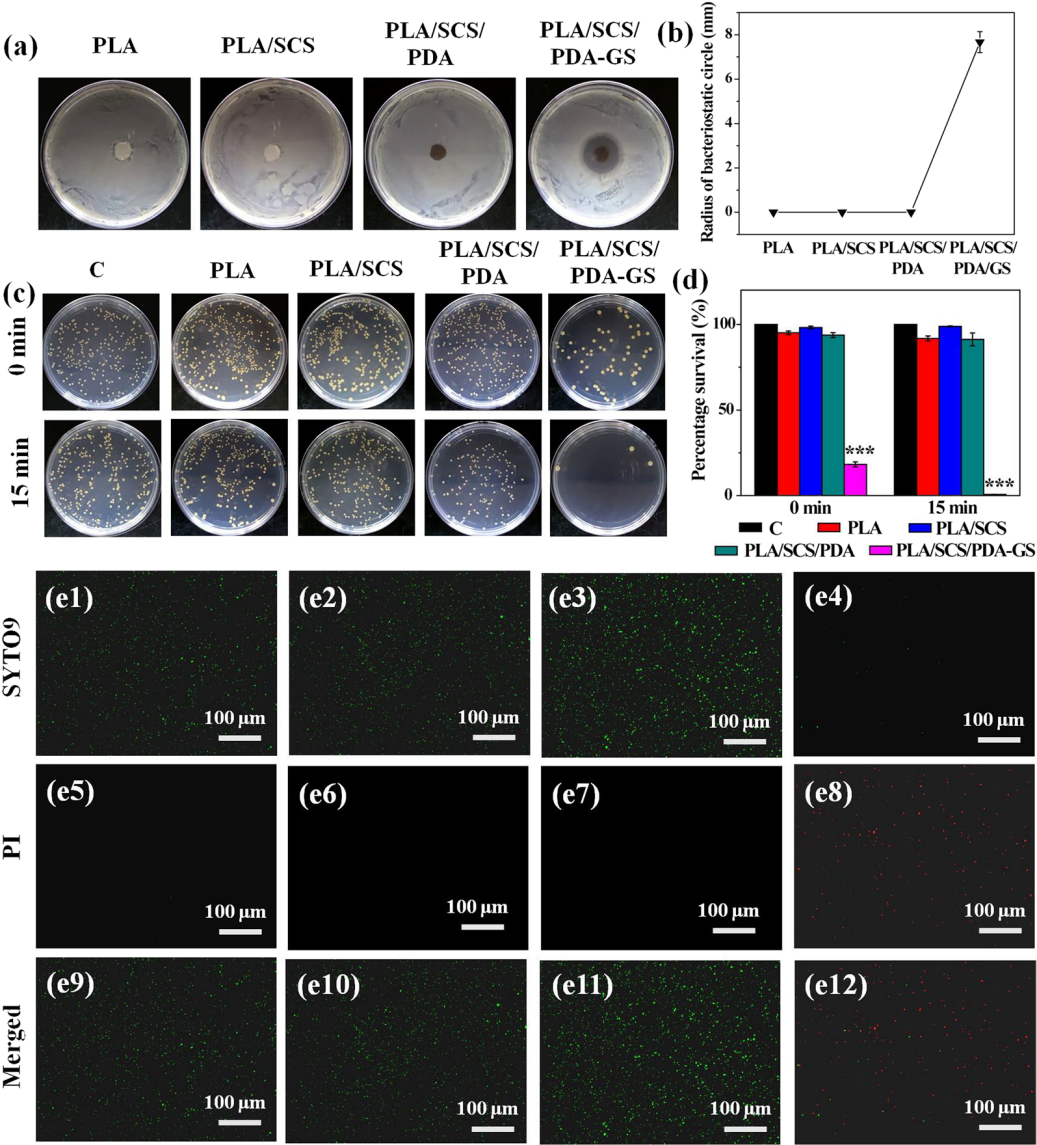
**a** Bacteriostatic circles and **b** radius of bacteriostatic circle of *S. aureus* after treatment with PLA, PLA/SCS, PLA/SCS/PDA and PLA/SCS/PDA-GS nanofiber membranes; **c** Photographs and **d** relative bacterial survival rates of *S. aureus* after coculture with PLA, PLA/SCS, PLA/SCS/PDA and PLA/SCS/PDA-GS nanofiber membranes for 0 min and 15 min respectively; Live/dead staining of S. aureus incubated with (**e1**, **e5**, **e9**) PLA, (**e2**, **e6**, **e10**) PLA/SCS, (**e3**, **e7**, **e11**) PLA/SCS/PDA and (**e4**, **e8**, **e12**) PLA/SCS/PDA-GS nanofiber membranes for 15 min (SYTO9 and PI stained live bacteria and dead bacteria, respectively)

Bacterial infection is a critical barrier to diabetic wound healing; therefore, antimicrobial activity is essential in diabetic wound dressings to prevent bacterial infection. Here, the *S. aureus* was used to investigate the in vitro antibacterial properties of the PLA-based nanofiber membrane. The ZOI assays showed no antibactericidal effects against *S. aureus* in PLA, PLA/SCS, and PLA/SCS/PDA groups, but for the PLA/SCS/PDA-GS nanofiber membranes, an 8 mm ZOI against *S. aureus* was observed (Fig. 8a, b). Additionally, the quantitative bactericidal effects from membranes were using through shake-flask culture method (Fig. 8c, d). Consistent with ZOI data, *S. aureus* had high survival rates when incubated with PLA, PLA/SCS, and PLA/SCS/PDA nanofiber membranes, but few *S. aureus* were observed in the PLA/SCS/PDA-GS treated group after co-cultivation for 15 min. It is worth noting that dopamine deposition had no obvious effects on *S. aureus* killing. Live/dead staining assays showed that bacteria grew well in PLA, PLA/SCS, and PLA/SCS/PDA groups, whereas GS-loaded nanofiber membranes (PLA/SCS/PDA-GS) showed efficient bacteria killing functions (99.23 ± 0.6%) (Fig. 8e1-e12). All the above results indicated that PLA, PLA/SCS and PLA/SCS/PDA nanofiber membranes had no significant anti-bactericidal abilities against *S. aureus*, whereas GS loading onto nanofiber membranes contributed to effective antibacterial activity.

## Conclusions

In summary, in the present work, a multifunctional nanofiber membrane was developed via electrospinning combined with surface modification methods. Importantly, the coating of SCS, PDA and GS changed the porous structure of PLA nanofiber, increased the PLA fiber diameter and improved the hydrophilicity property of PLA nanofiber membrane. Additionally, in vitro data confirmed anti-inflammatory properties of PLA/SCS/PDA-GS nanofiber membranes toward macrophages (Raw 264.7 cells) and increased endogenous VEGF secretion to induce vascularization. Moreover, our in vitro antibacterial data demonstrated the efficient antibacterial ability of the GS-loaded nanofiber membrane toward *S. aureus*. Therefore, our novel nanofiber membranes with antibacterial, anti-inflammatory, and angiogenic properties have promising potential in diabetic wound regeneration applications.

## Data Availability

All the data and materials concerned with the manuscript are available with the corresponding author and can thereby asked.
